# NLRX1 alleviates sepsis-induced acute lung injury by activating mitophagy and suppressing NLRP3 inflammasome activation

**DOI:** 10.3389/fphar.2026.1797066

**Published:** 2026-04-17

**Authors:** Zilong Hu, Jing Ding, Meng Huo, Yuqing Lu, Rui Wang, Liyuan Zhang, Dawei Li

**Affiliations:** 1 Department of Critical Care Medicine, The Sixth Medical Center, Chinese PLA General Hospital, Beijing, China; 2 Department of Pulmonary and Critical Care Medicine, The Sixth Medical Center, Chinese PLA General Hospital, Beijing, China

**Keywords:** acute lung injury, mitochondrial autophagy, NLRP3, NLRX1, sepsis

## Abstract

**Background:**

Sepsis-induced acute lung injury (ALI) is a life-threatening condition with limited therapeutic options. The mitochondrial protein NOD-like receptor X1 (NLRX1) has emerged as a potential immunometabolic modulator, but its functional role and mechanism in septic ALI remain poorly defined.

**Methods:**

Bioinformatic analysis was performed on the GSE4607 sepsis dataset. A murine model of sepsis-induced ALI was established using cecal ligation and puncture (CLP), with NLRX1 overexpression achieved through adeno-associated virus serotype 9 (AAV9)-mediated gene delivery. Histopathological evaluation, TUNEL staining, and transmission electron microscopy, ELISA were employed to assess lung injury. Mouse lung epithelial cells (MLE-12) were stimulated with lipopolysaccharide (LPS), combined with NLRX1 overexpression and Mdivi-1-mediated mitophagy inhibition to explore the key mechanism by which NLRX1 improves ALI.

**Results:**

NLRX1 was significantly downregulated in septic patients and mouse lungs, correlating with mitochondrial damage and NOD-like receptor protein 3 (NLRP3) inflammasome activation. NLRX1 overexpression in CLP mice attenuated pulmonary injury, edema, inflammation, and systemic cytokine release by enhancing mitophagy and suppressing apoptosis. Mechanistically, NLRX1 directly interacted with LC3B to promote mitophagy, thereby preserving mitochondrial membrane potential, reducing superoxide production and mtDNA release, and maintaining ATP levels. By improving mitochondrial homeostasis, NLRX1 overexpression indirectly suppressed NLRP3 inflammasome activation and pyroptosis. Crucially, the mitochondrial fission and mitophagy inhibitor Mdivi-1 abolished all beneficial effects of NLRX1, underscoring the essential role of comprehensive mitochondrial quality control.

**Conclusion:**

Our findings identify NLRX1 as a critical protective regulator of mitochondrial integrity that alleviates septic ALI by orchestrating mitophagy and mitochondrial quality control to restrain NLRP3-driven inflammation, presenting a promising therapeutic target.

## 1 Introduction

Sepsis-induced acute lung injury (ALI) remains a leading cause of mortality in intensive care units worldwide, with a mortality rate exceeding 40% despite advances in supportive care (Wick et al., 2024; Sun et al., 2023). As the first organ affected by systemic sepsis, the lungs are particularly vulnerable to the dysregulation of inflammatory cascades and mitochondrial dysfunction, which are hallmarks of this life-threatening syndrome (Zhu et al., 2024). The lack of targeted therapeutic strategies underscores the urgent need to uncover the molecular mechanisms underlying the pathogenesis of septic ALI (Qiao et al., 2024).

Mitochondrial autophagy (mitophagy), the selective degradation of damaged mitochondria via the autophagic pathway (Sciarretta et al., 2018). Mitochondrial homeostasis is a cornerstone of pulmonary epithelial and immune cell function, and its disruption is a hallmark of septic ALI (Larson et al., 2020; Faust et al., 2020). During sepsis, insufficient mitochondrial autophagy leads to significant mitochondrial dysfunction, characterized by disrupted electron transport chain activity, impaired oxidative phosphorylation, and excessive production of mitochondrial reactive oxygen species (mtROS), ultimately worsening ALI (Zhu et al., 2024; Han et al., 2025; Jiang et al., 2024). Defective mitophagy in sepsis leads to the accumulation of dysfunctional mitochondria, which in turn triggers the activation of the NOD-like receptor protein 3 (NLRP3) inflammasome (Peng et al., 2021). This a key driver of pro-inflammatory pyroptosis and cytokine release (Jiang et al., 2024; Liu et al., 2024). The NLRP3 inflammasome-mediated cleavage of gasdermin D (GSDMD) and secretion of interleukin (IL)-1β and IL-18 amplify the inflammatory response in the lung, exacerbating alveolar barrier disruption and tissue injury (Lee et al., 2016). While the interplay between mitophagy and NLRP3 inflammasome activation is increasingly recognized, the specific molecular regulators that coordinate these processes in septic ALI remain poorly defined.

NOD-like receptor X1 (NLRX1), a mitochondria-localized innate immune receptor, has emerged as a unique modulator of cellular stress responses and inflammatory signaling. NLRX1 has been implicated in regulating diverse cellular processes, including antiviral immunity, apoptosis, ROS generation, and mitochondrial metabolism (Bi et al., 2024). Accumulating evidence indicates that NLRX1 participates in the suppression of excessive inflammation by regulating mitochondrial function and antiviral immunity (Gill et al., 2025; Sá-Pessoa et al., 2023; Koo et al., 2021). NLRX1 can interact with autophagic machinery to modulate mitophagy in organ injury, cancer and viral infection models (Ding et al., 2024; Tao et al., 2024; Li et al., 2021; Peng et al., 2025; Allen et al., 2011; Singh et al., 2019). Given the critical role of mitophagy in preserving mitochondrial integrity and limiting inflammasome activation in ALI, we hypothesized that NLRX1 may act as a protective factor in septic ALI by enhancing mitophagy, thereby indirectly suppressing downstream NLRP3-driven inflammation rather than targeting the inflammasome directly.

In this study, we combined bioinformatic analysis of clinical sepsis datasets, *in vivo* murine models of cecal ligation and puncture (CLP)-induced septic ALI, and *in vitro* experiments using alveolar epithelial cells to elucidate the functional role and molecular mechanism of NLRX1. We demonstrate that NLRX1 is downregulated in septic patients and mouse lungs, and its overexpression attenuates pulmonary injury, edema, and inflammation by promoting mitophagy. These findings identify NLRX1 as a regulator of mitochondrial homeostasis and inflammasome signaling in septic ALI, offering a potential therapeutic target for this devastating condition.

## 2 Materials and methods

### 2.1 Animal models and experimental groups

All animal experiments were approved by the Ethics Committee of the Sixth Medical Center, Chinese PLA General Hospital (Approval No: 2023-x18-98). Male C57BL/6 mice (8–10 weeks old, weighing 20–25 g) were obtained from Vital River Laboratory Animal Technology Co., Ltd (Beijing, China). Mice were housed in a specific pathogen-free (SPF) facility under controlled conditions: temperature of 22 °C ± 2 °C, relative humidity of 50% ± 10%, and a 12-h light/12-h dark cycle. Standard laboratory chow and water were provided *ad libitum*. Animals were acclimatized to the environment for at least 1 week prior to experimentation.

A total of 36 mice were used in this study. Mice were randomly assigned to one of six experimental groups (n = 6 per group) using a computer-generated random number table: (1) Sham group; (2) CLP group; (3) Sham + AAV-Ctrl group; (4) Sham + AAV-NLRX1 group; (5) CLP + AAV-Ctrl group; (6) CLP + AAV-NLRX1 group. For NLRX1 overexpression, an adeno-associated virus serotype 9 (AAV9; Genechem, Shangha, China) vector carrying mouse NLRX1 cDNA under the control of the CMV promoter (AAV9-NLRX1) or an empty control vector (AAV9-Ctrl) was used. Four weeks prior to CLP or sham surgery, mice received a tail vein injection at a dose of 1 × 10^11^ viral genomes of AAV9-NLRX1 or AAV9-Ctrl (Liu et al., 2024). Four weeks post-injection (Day 0), the cecal ligation and puncture (CLP) model was established to induce polymicrobial sepsis (Liu et al., 2024). Briefly, mice were anesthetized with isoflurane inhalation, and a midline laparotomy was performed. The cecum was exposed, ligated distal to the ileocecal valve with a 4-0 silk suture, and punctured twice with a 21-gauge needle. A small amount of feces was extruded to ensure patency. The cecum was then returned to the abdominal cavity, and the incision was closed. Sham-operated mice underwent identical surgical procedures except for ligation and puncture. All animals were resuscitated with subcutaneous saline (1 mL) post-operatively. Animal health and behavior were monitored every 6 h post-surgery. Humane endpoints were defined as immobility, inability to access food/water, or severe respiratory distress. All mice were euthanized by carbon dioxide (CO_2_) inhalation at 24 h post-CLP to collect lung tissues and bronchoalveolar lavage fluid (BALF) for downstream analysis. No animals died prior to the planned endpoint in the Sham group; mortality in CLP groups prior to the endpoint was recorded but excluded from tissue analysis.

### 2.2 Collection and analysis of BALF

At 24 h post-CLP or sham surgery, mice were euthanized. The trachea was exposed and cannulated. The lungs were irrigated three times with 0.5 mL of ice-cold sterile phosphate-buffered saline (PBS). The recovered BALF was centrifuged at 1,000 *g* for 10 min at 4 °C. The supernatant was collected and stored at −80 °C for subsequent cytokine analysis. The protein concentrations in BALF were determined with BCA protein assay kit (Thermo Fisher Scientific, 23227, UNITED STATES). The cell pellet was resuspended in PBS, and total cell counts were determined using a hemocytometer (Zhu et al., 2024).

### 2.3 Lung wet-to-dry weight ratio measurement

Right lung lobes were weighed immediately (wet weight) and dried at 60 °C to constant weight (dry weight). The wet-to-dry ratio was calculated as wet weight/dry weight.

### 2.4. lung histopathology and hematoxylin and eosin (HE) staining

2.4

The left lung lobe was inflation-fixed with 4% paraformaldehyde via tracheal instillation, embedded in paraffin, and sectioned at 5-μm thickness. Sections were deparaffinized, rehydrated, and stained with HE according to standard protocols. Histopathological changes, including alveolar wall thickening, inflammatory cell infiltration, and hemorrhage, were evaluated by a pathologist blinded to the experimental groups. A semi-quantitative lung injury score was applied (Ji et al., 2024).

### 2.5 Transmission electron microscopy (TEM)

Fresh lung tissues (∼1 mm^3^) from each group were fixed in 2.5% glutaraldehyde overnight at 4 °C. After washing with PBS, samples were post-fixed in 1% osmium tetroxide, dehydrated through a graded ethanol series, and embedded in epoxy resin. Ultrathin sections (70 nm) were cut, stained with uranyl acetate and lead citrate, and examined under a transmission electron microscope (Hitachi, Japan) to observe mitochondrial ultrastructure and autophagic vesicles.

### 2.6 Terminal deoxynucleotidyl transferase dUTP nick end labeling (TUNEL) staining

Apoptosis in lung tissues was detected using a TUNEL assay kit (Thermo Fisher Scientific, C10618, United States) according to the manufacturer’s instructions. Briefly, paraffin-embedded lung sections were deparaffinized and permeabilized with proteinase K. After incubation with the TUNEL reaction mixture, the sections were counterstained with DAPI. TUNEL-positive (apoptotic) cells were visualized and counted under a fluorescence microscope (Olympus, IX83, Japan).

### 2.7 Cell culture and In vitro model

Mouse lung epithelial cells (MLE-12) were obtained from ATCC and cultured in DMEM/F12 medium (Gibco, United States) supplemented with 10% fetal bovine serum and 1% penicillin/streptomycin at 37 °C in a 5% CO_2_ atmosphere. To establish an *in vitro* model of acute lung injury, cells were treated with lipopolysaccharide (LPS, 1 μg/mL, Sigma-Aldrich, L2630, United States) for 24 h. For NLRX1 overexpression, cells were transfected with an NLRX1-expressing lentiviral plasmid virus (GeneCopoeia, EX-Mm29688-Lv105, China) or empty vector virus (GeneCopoeia, EX-NEG-Lv105, China) prior to LPS stimulation. For mitophagy inhibition experiments, cells were treated with Mdivi-1 (Sigma-Aldrich, M0199, United States) at a concentration of 10 μM for 2 h prior to LPS stimulation.

### 2.8 Western blot analysis

Protein lysates from lung tissues and cells were separated by sodium dodecyl sulfate-polyacrylamide gel electrophoresis (Bio-Rad, 4561083, United States), transferred to PVDF membranes (Millipore, IPVH00010, United States), and probed with primary antibodies against NLRX1 (1:1,000, Abcam, ab307668, United Kingdom), NLRP3 (1:1,000, Abcam, ab263899, United Kingdom), GSDMD-N (1:1,000, Abcam, ab209845, United Kingdom), Cleaved caspase-1 (1:1,000, Cell Signaling Technology, 89332, United States), LC3B (1:1,000, Cell Signaling Technology, 4,108, United States), p62 (1:1,000, Abcam, ab109012, United Kingdom), and GAPDH (1:2000, Abcam, ab9485, United Kingdom). HRP-conjugated secondary antibodies (1:5,000) were used for detection. Band intensities were quantified using ImageJ (version 1.46r; NIH, Bethesda, United States).

### 2.9 Enzyme-linked Immunosorbent assay (ELISA)

The concentrations of IL-1β (MLB00C), tumor necrosis factor (TNF)-α (MTA00B), IL-6 (M6000B), and IL-18 (7,625) in BALF, serum, and cell culture supernatants were quantified using commercial ELISA kits (R&D Systems, United States) according to the manufacturers’ protocols. Absorbance was measured at 450 nm on a microplate reader (BioTek, United States), and cytokine concentrations were calculated from standard curves.

### 2.10 Cellular Immunofluorescence (IF)

MLE-12 cells grown on glass coverslips were fixed with 4% paraformaldehyde (PFA, Sigma-Aldrich, 158127, United States) permeabilized with 0.1% Triton X-100, and blocked with 5% BSA. Cells were then incubated with primary antibodies against NLRX1, LC3B, and TOMM20 overnight at 4 °C, followed by incubation with appropriate fluorescently labeled secondary antibodies (e.g., Alexa Fluor 488, 555, or 647). Nuclei were counterstained with DAPI. Images were captured using a confocal laser scanning microscope (Leica, Wetzlar, Germany), and co-localization was analyzed.

### 2.11 Measurement of mitochondrial membrane potential (ΔΨm)

The ΔΨm in MLE-12 cells was assessed using the fluorescent dye JC-1 (Thermo Fisher Scientific, T3168, United States) as described (Zhou et al., 2017). According to the protocol, cells were incubated with JC-1 staining solution (5 μg/mL) at 37 °C for 20 min. In healthy cells with high ΔΨm, JC-1 forms aggregates emitting red fluorescence; in depolarized cells, it remains as monomers emitting green fluorescence. The red/green fluorescence intensity ratio was quantified using a confocal microscopy (Leica, Wetzlar, Germany).

### 2.12 Detection of mitochondrial superoxide (MitoSOX)

Mitochondrial superoxide levels were measured using MitoSOX™ Red (Thermo Fisher Scientific, M36008, United States), a mitochondrial superoxide indicator. MLE-12 cells were loaded with MitoSOX™ Red (5 μM) and incubated at 37 °C for 30 min. After washing, the fluorescence intensity was measured by fluorescence microscopy (Olympus, IX83, Japan).

### 2.13 Cellular ATP content assay

Intracellular ATP levels were determined using an ATP assay kit based on the luciferin-luciferase reaction (Beyotime, S0026, China). Briefly, MLE-12 cells were lysed, and the lysates were centrifuged. The supernatant was mixed with the ATP detection working solution, and luminescence was immediately measured with a luminometer. ATP concentrations were calculated based on a standard curve and normalized to the total protein concentration.

### 2.14 Statistical analysis

All data are presented as mean ± standard deviation (SD). Statistical analyses were performed using GraphPad Prism software (GraphPad, San Diego, CA, United States). Comparisons between two groups were analyzed by unpaired Student’s t-test. For multiple group comparisons, one-way analysis of variance (ANOVA) followed by Tukey’s post-hoc test was applied. A P-value of less than 0.05 was considered statistically significant.

## 3 Results

### 3.1 NLRX1 expression is downregulated in sepsis and correlates with mitochondrial damage and NLRP3 inflammasome activation

**FIGURE 1:**
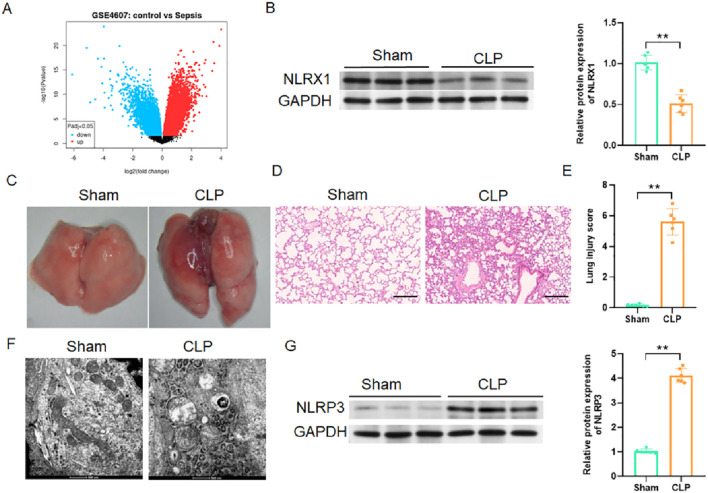
NLRX1 deficiency in experimental and clinical sepsis correlates with mitochondrial dysfunction and NLRP3 inflammasome activation. **(A)** Transcriptomic profiling of the GSE4607 dataset demonstrates significant downregulation of NLRX1 expression in whole blood from septic patients (n = 69) compared to healthy controls (n = 15). **(B–E)** Validation in a murine CLP model: **(B)** Representative western blots and quantification of NLRX1 protein expression in lung tissues (n = 6/group). **(C,D)** Representative lung morphological images and HE-stained lung sections (scale bar: 100 μm). **(E)** Semiquantitative lung injury scores showing aggravated histopathological alterations in CLP mice (n = 6/group). **(F)** TEM of lung tissues (scale bar: 500 nm) reveal disrupted mitochondrial cristae and aberrant mitophagic activity in CLP-operated mice. **(G)** Immunoblot analysis of NLRP3 shows enhanced activation in septic lungs (n = 6/group). **p < 0.01 vs. Sham group.

Bioinformatic analysis of the GSE4607 dataset revealed a significant downregulation of NLRX1 in whole blood from septic patients compared to healthy controls (Figure 1A). This clinical observation was confirmed in a mouse model of sepsis-induced acute lung injury. The expression of NLRX1 protein in lung tissue was significantly decreased after CLP, which was consistent with the characteristic histopathological injuries such as inflammatory infiltration, edema, and thickening of alveolar walls (Figure 1B-E). Given the mitochondrial localization of NLRX1, we investigated mitochondrial ultrastructure and found that CLP induced severe damage, manifested by disrupted cristae and morphological changes indicative of aberrant mitophagy (Figure 1F). Furthermore, this milieu of NLRX1 deficiency and mitochondrial dysfunction was associated with a robust activation of the NLRP3 inflammasome in the injured lungs (Figure 1G). These findings collectively position NLRX1 downregulation may be a key event linking septic insult to mitochondrial destabilization and pro-inflammatory signaling in the lung.

### 3.2 NLRX1 overexpression attenuates pulmonary injury and systemic inflammation in murine sepsis

**FIGURE 2:**
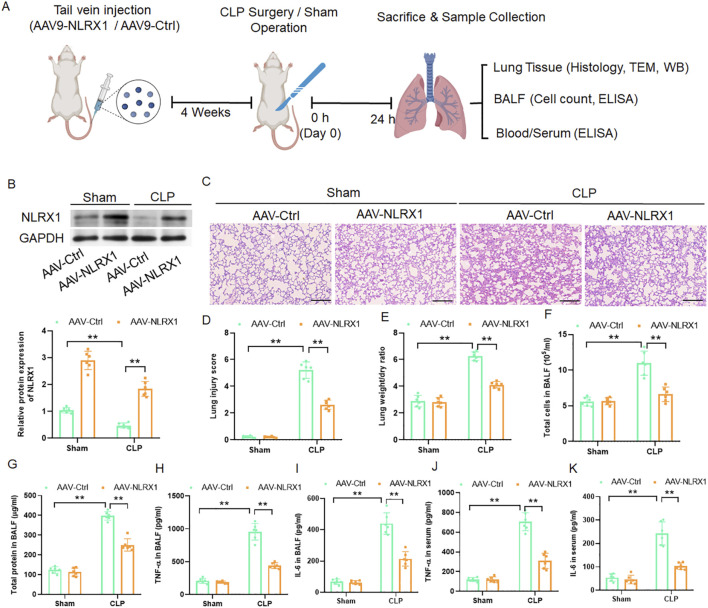
Pulmonary-specific overexpression of NLRX1 ameliorates sepsis-induced lung injury and systemic inflammation. **(A)** Representative immunoblots and quantitative analysis confirming successful NLRX1 overexpression in lung tissues of AAV-NLRX1 treated mice (n = 6/group). **(B)** Representative HE-stained lung sections (scale bar: 100 μm) showing improved pulmonary architecture in NLRX1-overexpressing mice following CLP. **(C)** Semiquantitative assessment of lung injury scores demonstrates significant histological improvement in AAV-NLRX1 group (n = 6/group). **(D)** Lung wet/dry weight ratio measurement reveals attenuated pulmonary edema in NLRX1-overexpressing mice (n = 6/group). **(E,F)** Bronchoalveolar lavage fluid analysis shows reduced total cell counts **(E)** and protein concentration **(F)** in AAV-NLRX1 treated mice (n = 6/group). **(G–J)** Cytokine profiling indicates suppressed levels of pro-inflammatory mediators TNF-α and IL-6 in both BALF **(G,H)** and serum **(I, J)** of NLRX1-overexpressing mice (n = 6/group). **p < 0.01 vs. AAV-Ctrl + sham group or AAV-Ctrl + CLP group.

To investigate the therapeutic potential of NLRX1, we employed an AAV vector to achieve NLRX1 overexpression. Immunoblotting analyses confirmed robust transgene expression of NLRX1 in the lungs of AAV-NLRX1-treated mice (Figure 2A). Histopathological evaluation revealed that NLRX1 overexpression markedly mitigated the structural damage characteristic of sepsis-induced ALI, as evidenced by significantly diminished inflammatory cell infiltration, attenuated alveolar wall thickening, and a consequent reduction in the overall lung injury score compared to the CLP group receiving the control vector (Figures 2B,C). Furthermore, the elevation in lung wet-to-dry ratio, a key indicator of pulmonary edema, was substantially reversed by NLRX1 augmentation (Figure 2D). Analysis of BALF demonstrated that the protective effects of NLRX1 extended to the alveolar space, with a pronounced reduction in both total inflammatory cell count and protein concentration (Figures 2E,F). Importantly, NLRX1 overexpression also suppressed the systemic inflammatory response, as indicated by significantly lower levels of the pro-inflammatory cytokines TNF-α and IL-6 not only in BALF but also in the serum (Figures 2G–J). Collectively, these data demonstrate that targeted restoration of NLRX1 expression in the lung could effectively preserve pulmonary architecture and dampen local and systemic inflammation in experimental sepsis.

### 3.3 NLRX1 overexpression enhances mitophagy and suppresses apoptosis in septic lungs

**FIGURE 3:**
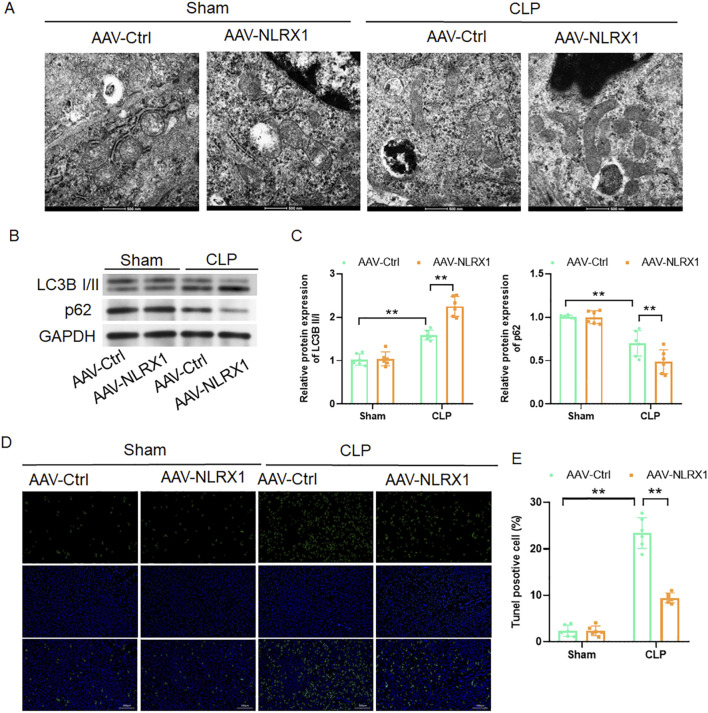
NLRX1 overexpression enhances mitophagic activity and reduces cellular apoptosis in septic lungs. **(A)** Representative transmission electron micrographs of lung tissues (scale bar: 500 nm) showing increased formation of double-membrane autophagic structures encapsulating mitochondria in AAV-NLRX1 treated mice compared to controls. **(B,C)** Western blot analysis of autophagy markers: **(B)** Representative immunoblots of LC3B I/II and p62; **(C)** Quantitative analysis demonstrating elevated LC3B II/I ratio and reduced p62 accumulation in NLRX1-overexpressing mice (n = 6/group). **(D,E)** TUNEL assay for apoptosis detection: **(D)** Representative fluorescent images of TUNEL-positive cells (green) with DAPI nuclear counterstain (blue) (scale bar: 200 μm); **(E)** Quantification of apoptotic cell ratio showing significant reduction in AAV-NLRX1 group (n = 6/group). **p < 0.01 vs. AAV-Ctrl + sham group or AAV-Ctrl + CLP group.

Having established the protective effects of NLRX1 on pulmonary pathology and inflammation, we sought to delineate the underlying cellular mechanisms, with a focus on mitochondrial quality control and cell survival. TEM of lung tissue from AAV-NLRX1-treated septic mice revealed a significant increase in the presence of mitochondria encapsulated within double-membrane structures, indicative of enhanced mitophagic activity (Figure 3A). This morphological evidence was corroborated at the molecular level by immunoblot analysis, which demonstrated that NLRX1 overexpression significantly elevated the LC3B II/I ratio while concurrently reducing the abundance of the autophagic substrate p62 (Figures 3B,C), consistent with accelerated autophagic activity. Furthermore, TUNEL staining revealed a marked decrease in the proportion of apoptotic cells in the lungs of the AAV-NLRX1 group compared to the septic controls (Figures 3D,E). These findings collectively indicate that NLRX1-mediated protection against sepsis-induced lung injury is mechanistically linked to the promotion of mitochondrial clearance through autophagy and the subsequent attenuation of cellular apoptosis.

### 3.4 NLRX1 overexpression blunts NLRP3 inflammasome activation and pyroptotic signaling in the lung

**FIGURE 4:**
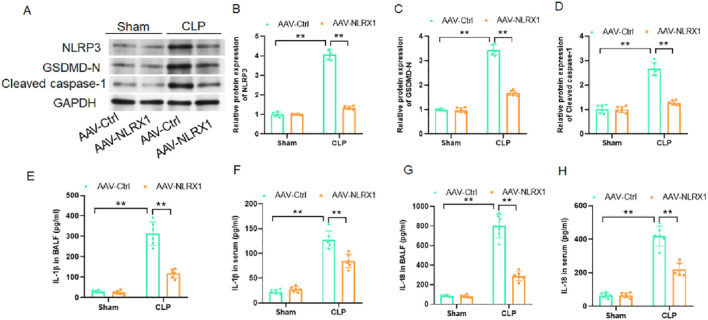
NLRX1 overexpression suppresses NLRP3 inflammasome activation and pyroptotic signaling in septic lung injury. **(A–D)** Analysis of NLRP3 inflammasome components: **(A)** Representative western blots of NLRP3, GSDMD-N, and cleaved caspase-1; **(B–D)** Densitometric quantification demonstrates significant reduction in protein levels of NLRP3 **(B)**, GSDMD-N **(C)**, and cleaved caspase-1 **(D)** in lung tissues of AAV-NLRX1 treated mice (n = 6/group). **(E–H)** Cytokine measurement in biological fluids: ELISA analysis shows attenuated concentrations of IL-1β and IL-18 in both BALF **(E,F)** and serum **(G,H)** from AAV-NLRX1 group compared to controls (n = 6/group). **p < 0.01 vs. AAV-Ctrl + sham group or AAV-Ctrl + CLP group.

To elucidate the mechanism by which NLRX1 confers protection against septic injury, we investigated its impact on the NLRP3 inflammasome pathway and the ensuing pyroptotic cell death. Western blot analysis of lung homogenates demonstrated that NLRX1 overexpression significantly suppressed sepsis-induced activation of this pathway. Specifically, protein levels of the core inflammasome component NLRP3, the pyroptosis executioner protein GSDMD-N terminal fragment (GSDMD-N), and the activated form of caspase-1 (cleaved caspase-1) were all markedly reduced in AAV-NLRX1 treated mice compared to the CLP control group (Figures 4A–D). Consistent with the inhibition of inflammasome activity, the downstream release of its canonical cytokines, IL-1β and IL-18, was profoundly attenuated. ELISA measurements confirmed significantly lower concentrations of both IL-1β and IL-18 in both BALF and serum from the AAV-NLRX1 group (Figures 4E–H). These results indicate that the protective effects of NLRX1 are mediated, at least in part, through inhibition of the NLRP3 inflammasome cascade and its associated pyroptotic and inflammatory signaling.

### 3.5 NLRX1 directly interacts with LC3B to promote mitophagic activity in LPS-induced MLE-12 cells

**FIGURE 5:**
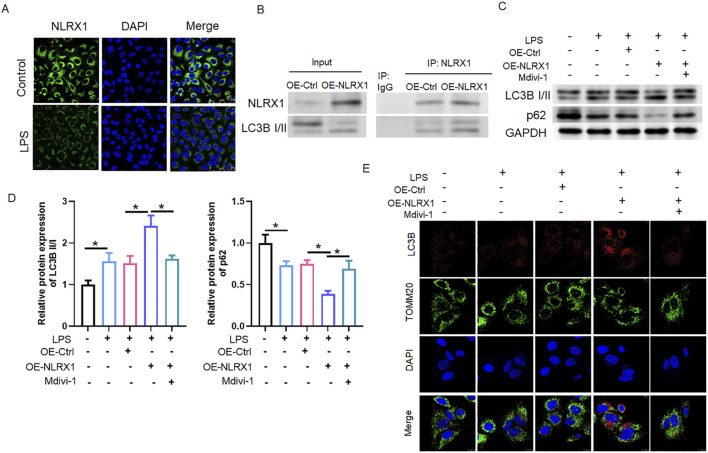
NLRX1 interacts with LC3 to promote mitophagy in MLE-12 cells. **(A)** Immunofluorescence analysis showing decreased NLRX1 expression (green) in LPS-stimulated MLE-12 cells compared to controls. Nuclei are stained with DAPI (blue) (Scale bar: 75 μm). **(B)** Co-immunoprecipitation assay demonstrating enhanced physical interaction between NLRX1 and LC3B in cells overexpressing NLRX1. **(C,D)** Western blot analysis of autophagy markers: **(C)** Representative immunoblots; **(D)** Quantitative analysis showing increased LC3B-II conversion and decreased p62 levels in NLRX1-overexpressing cells, effects that were abolished by Mdivi-1 treatment. **(E)** Confocal microscopy images and quantification of LC3B (red) and TOMM20 (green) co-localization (Scale bar: 10 μm). *p < 0.05 vs. Control, LPS, LPS + OE-Ctrl or LPS + OE-NLRX1 group.

To further investigate the mechanism by which NLRX1 alleviates ALI, we established an *in vitro* model using MLE-12 alveolar epithelial cells challenged with LPS. Immunofluorescence analysis confirmed that LPS stimulation downregulated NLRX1 expression (Figure 5A), mirroring our *in vivo* findings. We then investigated the functional interplay between NLRX1 and the autophagic machinery. Co-immunoprecipitation assays in MLE-12 cells revealed that NLRX1 overexpression significantly strengthened its physical interaction with LC3B I/II (Figure 5B), suggesting a direct role in recruiting the core autophagy component to facilitate mitochondrial targeting. The functional consequence of this interaction was a pronounced enhancement of mitophagy, as indicated by increased LC3B-II conversion and p62 degradation, which were effectively abolished by the mitochondrial fission and mitophagy inhibitor Mdivi-1 (Figures 5C,D). Furthermore, confocal microscopy demonstrated that LPS challenge increased the co-localization of LC3B with the mitochondrial marker TOMM20, an effect that was potentiated by NLRX1 overexpression. Crucially, this enhanced co-localization was abrogated in the presence of Mdivi-1 (Figure 5E). Collectively, these data establish that NLRX1 functions as a critical scaffold, directly engaging with LC3B to drive targeted mitophagy in lung epithelial cells, and that its cytoprotective role is dependent on this mitochondrial dynamic pathway.

### 3.6 NLRX1 preserves mitochondrial functional integrity in a mitophagy-dependent manner

**FIGURE 6:**
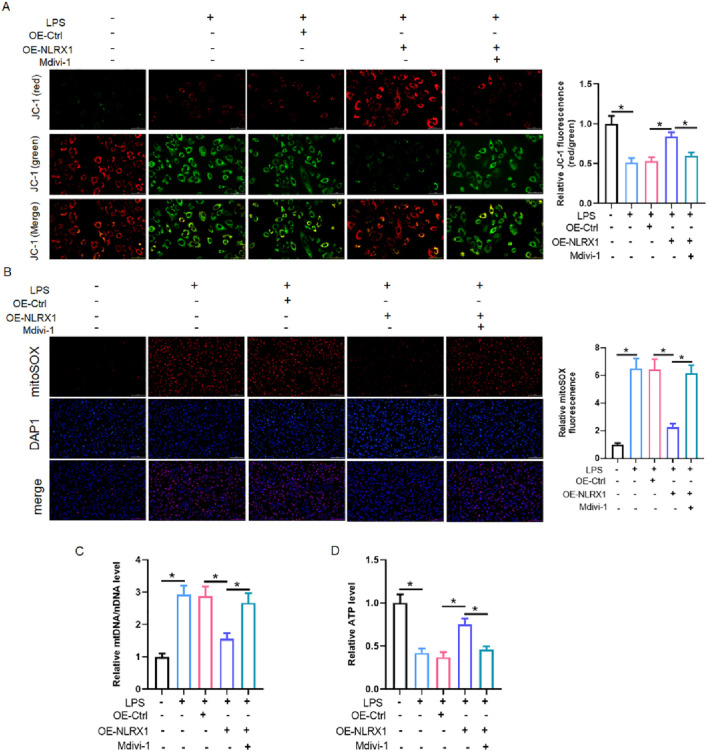
NLRX1 preserves mitochondrial function through mitophagy-dependent mechanisms *in vitro*. **(A)** Assessment of mitochondrial membrane potential using JC-1 staining (Scale bar: 50 μm). **(B)** Measurement of mitochondrial superoxide production using MitoSOX™ Red (Scale bar: 200 μm). **(C)** Quantitative analysis of cytosolic mtDNA levels by qPCR. **(D)** Cellular ATP content measurement. *p < 0.05 vs. Control, LPS, LPS + OE-Ctrl or LPS + OE-NLRX1 group.

To determine whether NLRX1-mediated protection involves direct modulation of mitochondrial function, we performed a comprehensive assessment of mitochondrial parameters in LPS-challenged MLE-12 cells. LPS stimulation triggered severe mitochondrial dysfunction, characterized by a collapse of theΔΨm, a surge in mitochondrial superoxide production, elevated cytosolic mtDNA levels, and a significant reduction in cellular ATP content (Figures 6A–D). Notably, NLRX1 overexpression effectively reversed all these pathological alterations, restoring ΔΨm, quenching mitochondrial superoxide, curtailing mtDNA release, and rescuing cellular bioenergetics (Figures 6A–D). To establish a causal link between these functional benefits and NLRX1-driven mitophagy, we employed the inhibitor Mdivi-1. Strikingly, the presence of Mdivi-1 completely abrogated the beneficial effects of NLRX1 on all measured parameters of mitochondrial function (Figures 6A–D). These results demonstrate that NLRX1 may be essential for maintaining mitochondrial functional integrity under inflammatory stress and that this protection requires intact mitochondrial dynamics and quality control mechanisms, including but not limited to mitophagy.

### 3.7 NLRX1-mediated mitophagy attenuates NLRP3 inflammasome activation via preservation of mitochondrial homeostasis

**FIGURE 7:**
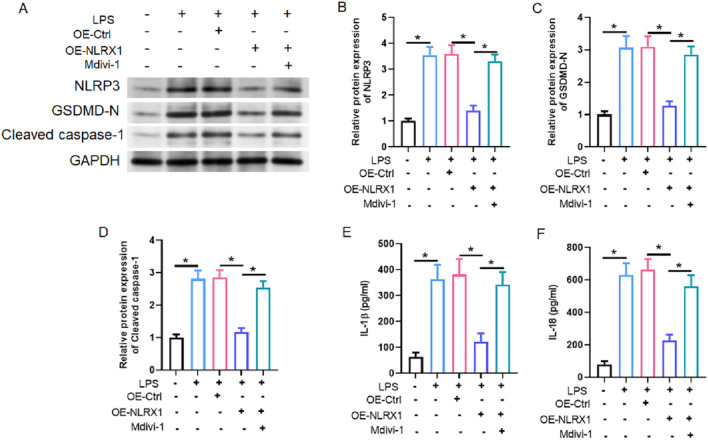
NLRX1 suppresses NLRP3 inflammasome activation indirectly through preservation of mitochondrial integrity. **(A)** Representative western blots of NLRP3, GSDMD-N, and cleaved caspase-1; **(B–D)** Quantitative analysis demonstrates that NLRX1 overexpression significantly reduces protein levels of NLRP3 **(B)**, GSDMD-N **(C)**, and cleaved caspase-1 **(D)** in LPS-stimulated MLE-12 cells, while Mdivi-1 co-treatment abolishes these inhibitory effects. **(E,F)** ELISA measurements show that NLRX1 overexpression attenuates LPS-induced release of mature IL-1β **(E)** and IL-18 **(F)** into cell culture supernatants, effects that are reversed by mitophagy inhibition with Mdivi-1. *p < 0.05 vs. Control, LPS, LPS + OE-Ctrl or LPS + OE-NLRX1 group.

To establish the functional link between NLRX1-driven mitophagy and inflammasome regulation, we examined the activation status of the NLRP3 pathway in our *in vitro* model. LPS stimulation of MLE-12 cells robustly activated the NLRP3 inflammasome, as demonstrated by significantly elevated protein levels of NLRP3, the pyroptosis executor GSDMD-N, and cleaved caspase-1, along with increased secretion of the mature cytokines IL-1β and IL-18 (Figures 7A–E). NLRX1 overexpression effectively suppressed the activation of this entire pathway. Crucially, this anti-inflammatory effect was entirely dependent on mitochondrial quality control and mitophagy, as co-treatment with Mdivi-1 completely rescinded the ability of NLRX1 to attenuate inflammasome component expression and cytokine production (Figures 7A–E). These data conclusively demonstrate that the potent anti-inflammatory activity of NLRX1 is not merely correlative but is mechanistically executed through its capacity to maintain mitochondrial fitness via mitophagy, thereby removing the fundamental triggers for NLRP3 inflammasome assembly and activation.

## 4 Discussion

The present study delineates a protective role of NLRX1 in septic ALI, demonstrating that its downregulation in clinical sepsis and experimental models correlates with impaired mitophagy, mitochondrial damage, and NLRP3 inflammasome hyperactivation. NLRX1 directly interacts with LC3B through its LIR motif to promote mitophagy, which in turn suppresses NLRP3 inflammasome activation by removing damaged mitochondria. These findings highlight NLRX1 as a potential therapeutic target in septic ALI.

The protective function of NLRX1 against excessive inflammation has been demonstrated in several sepsis-related models. In macrophages, NLRX1 downregulates IL-1β expression by interacting with IKK-α, thereby limiting tissue damage caused by excessive inflammatory responses during sepsis (Chen et al., 2022). In an LPS-induced lethal endotoxemia sepsis model, NLRX1-rich leucine repeat domain-treated mice exhibited reduced susceptibility alongside decreased IL-6 production, suggesting its negative regulatory role in the NF-κB signaling pathway (Koo et al., 2021). Knockout of NLRX1 suppressed mitochondrial autophagy in microglia and caused severe sepsis in the mouse brain (Peng et al., 2022). Our results demonstrated that NLRX1 was significantly downregulated in both septic patients (GSE4607 dataset), CLP-induced mouse lungs and LPS-induced MLE-12 cells. AAV9-mediated pulmonary-specific overexpression of NLRX1 was effective in attenuating lung injury, edema, and systemic inflammation, promoting mitophagy, reducing mitochondrial damage and NLRP3 inflammasome hyperactivation in CLP-induced ALI mice.

Most notably, a recent groundbreaking study identified NLRX1 as a novel mitophagy receptor that contains a conserved LC3B-interacting region (LIR) motif (Zhang et al., 2019; Killackey et al., 2023). Upon cellular stress, such as bacterial infection, NLRX1 undergoes oligomerization, exposing its LIR domain to facilitate binding to LC3B and thereby initiating mitophagy (Zhang et al., 2019; Killackey et al., 2023). Reports indicate that NLRX1 modulates mitochondrial autophagy and mitochondrial function in various diseases, thereby improving disease progression. For instance, in nucleus pulposus (NP) cells, NLRX1 promotes mitochondrial quality by regulating mitochondrial autophagy activity, thereby restoring disc vitality (Song et al., 2024). Zhang et al. found that disrupting NLRX1 eliminates cytoplasmic AcCoA reduction-induced mitochondrial autophagy, providing evidence for a metabolic mechanism underlying KRAS inhibitor resistance (Zhang et al., 2025). In renal ischemia-reperfusion injury, overexpression of NLRX1 induces LC3B lipidation to activate mitochondrial autophagy, thereby reducing inflammation and improving renal injury (Liao et al., 2024). In intestinal ischemia/reperfusion injury, NLRX1-dependent mitochondrial autophagy activation attenuates mitochondrial dysfunction and intestinal injury (Li et al., 2021). During ALI, activated mitochondrial autophagy facilitates the clearance of damaged mitochondria, restores mitochondrial function, and reduces oxidative stress and inflammation, thereby helping to mitigate the severity of ALI (Zhu et al., 2024; Zhao et al., 2021; Tang et al., 2023; Ornatowski et al., 2020). Given the interactions between NLRX1 and mitochondrial autophagy mechanisms described in the aforementioned literature, this suggests that NLRX1 may regulate mitochondrial autophagy in alveolar epithelial cells during ALI. Following overexpression of NLRX1, we observed that NLRX1 alleviates septic ALI through a mitochondria-autophagy-dependent mechanism. It directly interacts with LC3B to enhance mitochondrial clearance, maintain mitochondrial homeostasis, and ultimately suppress NLRP3-driven pyroptosis and inflammation. Furthermore, we utilized Mdivi-1 to antagonize the protective effects of NLRX1 *in vivo*. While often employed to block mitophagy, Mdivi-1 is primarily an inhibitor of DRP1-mediated mitochondrial fission. Complete reversal of NLRX1’s protective effects by Mdivi-1 further underscores that intact mitochondrial dynamics are essential for NLRX1 function.

Mitochondrial damage is a well-established trigger for NLRP3 activation, as dysfunctional mitochondria release ROS and mtDNA that act as DAMPs to initiate inflammasome assembly. These substances continuously and strongly activate the NLRP3 inflammasome, leading to extensive cleavage of Caspase-1, which in turn promotes the maturation and release of potent pro-inflammatory factors such as IL-1β and IL-18, triggering pyroptosis in alveolar epithelial cells and pulmonary vascular endothelium, damaging the blood-gas barrier, and causing pulmonary edema and respiratory failure. Accumulating evidence indicates that activating mitochondrial autophagy during ALI can attenuate NLRP3 inflammasome activation and improve lung injury (Jiang et al., 2024; Wu et al., 2021; Yan et al., 2025; White et al., 2022). Furthermore, in a model of chronic obstructive pulmonary disease (COPD), NLRX1 knockout mice produced more IL-1β and IL-18, indicating a potential role in inflammasome regulation (Kang et al., 2015). NLRX1 has been demonstrated to possess the ability to negatively regulate the NLRP3 inflammasome (Li et al., 2016; Tong et al., 2020). In a cerebral ischemia/reperfusion injury model, overexpression of NLRX1 induces microglial autophagy, thereby inhibiting NLRP3 inflammasome signaling and improving neurological damage (Peng et al., 2025). In LPS-induced cardiac injury, silencing NLRX1 exacerbates mitochondrial damage, thereby activating NLRP3 inflammasomes and leading to further worsening of cardiac injury (Zhao et al., 2020). Our data showed that NLRX1 overexpression reduced NLRP3, cleaved caspase-1, and GSDMD-N levels in septic lungs and LPS-stimulated MLE-12 cells, with concurrent decreased in IL-1β and IL-18 secretion. Although previous studies have established that autophagy alleviates mitochondrial DAMP-induced ALI (Peng et al., 2021), the specific upstream regulators remain elusive. Unlike prior reports suggesting NLRX1 mitigates sepsis via NF-κB regulation (Koo et al., 2021) or MAVS-dependent pathways in the heart (Li et al., 2016), our findings uncover a distinct mechanism in septic ALI: NLRX1 functions primarily through a direct interaction with LC3B to enhance mitophagy. This is evidenced by our finding that the mitophagy inhibitor Mdivi-1 completely negates the protective effects of NLRX1, demonstrating that NLRX1 does not directly target the NLRP3 inflammasome but instead suppresses its activation indirectly by resolving mitochondrial damage through mitophagy. If NLRX1 were a direct NLRP3 inhibitor, Mdivi-1 would not have reversed its anti-inflammatory effects. This finding clarifies the hierarchical relationship between mitochondrial quality control and inflammasome regulation in septic ALI, emphasizing that restoring mitophagy may be a more effective strategy to dampen inflammation than directly targeting the NLRP3 pathway.

Despite these promising findings, our study has several limitations that warrant discussion. First, although we identified NLRX1 downregulation in sepsis using the GSE4607 dataset, we did not validate these findings in a large, prospective clinical cohort. Future studies collecting serum and BALF samples from septic patients are necessary to confirm the diagnostic and prognostic value of NLRX1. Second, our mechanistic experiments primarily focused on MLE-12 and whole-lung overexpression. Future investigations utilizing cell-type-specific knockout or overexpression mouse models (e.g., targeting macrophages or endothelial cells) would provide deeper insights. Finally, regarding clinical translation, it is important to emphasize that our use of AAV-mediated NLRX1 overexpression serves as a mechanistic proof-of-concept rather than a directly translatable therapeutic modality. Future translational efforts should prioritize the identification and validation of fast-acting, small-molecule NLRX1 agonists or pharmacological modulators.

In summary, this study establishes NLRX1 as a key endogenous protective factor in sepsis-induced ALI. It achieves this by promoting LC3B-mediated mitophagy and maintaining mitochondrial quality control, thereby inhibiting NLRP3 inflammasome activation. These findings not only deepen our understanding of the immunometabolic regulatory network but also provide a theoretical basis for developing potential intervention strategies targeting NLRX1.

## Data Availability

The original contributions presented in the study are included in the article/supplementary material, further inquiries can be directed to the corresponding author.
